# Diagnosis of Acute Mesenteric Ischemia in a Patient with End-Stage Renal Disease with Normal Serum Lactate

**DOI:** 10.7759/cureus.6708

**Published:** 2020-01-20

**Authors:** John Taylor, Bayarmaa Mandzhieva, Rima Shobar

**Affiliations:** 1 Internal Medicine, AdventHealth, Orlando, USA

**Keywords:** acute mesenteric ischemia, pneumatosis, normal lactic acid, end-stage renal disease

## Abstract

Acute mesenteric ischemia (AMI) is a life-threatening vascular emergency and a diagnostic challenge for physicians. It represents a group of pathophysiologic processes that have a common end point, that is, bowel infarction, and has a nonspecific clinical picture and a high mortality rate. The most common underlying etiologies are arterial embolism, arterial thrombosis, nonocclusive mesenteric ischemia (NOMI), and mesenteric venous thrombosis. NOMI is caused by prolonged functional vasoconstriction of the visceral arterial vessels, leading to progressive intestinal ischemia, and could be defined by the absence of atherosclerotic thrombotic or embolic occlusion of the mesenteric arteries. The pathophysiology of NOMI remains poorly understood. It can occur in a wide range of critical systemic illnesses associated with hypotension and hypovolemia and in patients on hemodialysis. Elevation of serum lactate may not be present in a significant portion of patients with AMI, which creates an additional obstacle to the prompt diagnosis and often delays treatment. We present a case of a 35-year-old female with HIV and end-stage renal disease on hemodialysis who was admitted for complaints of vague nonspecific abdominal pain. Her initial lactic acid was normal, and due to renal function, she underwent CT of the chest and abdomen without contrast, which only revealed findings consistent with chronic constipation. She later developed vasopressor-dependent hypotension, but her serial lactic acids were all normal. Finally, CT of the abdomen and pelvis was performed with IV contrast, with findings of enterocolitis of the ileum and proximal ascending colon. There was also evidence of pneumatosis involving dilated loops of the small bowel in the lateral mid-abdomen. She underwent an exploratory laparotomy and was found to have gangrene of terminal ileum with associated perforation. Small bowel resection was performed, after which the patient clinically stabilized.

## Introduction

Acute mesenteric ischemia (AMI) is a life-threatening condition caused by a reduction of mesenteric blood flow with bowel ischemia and eventual gangrene of the bowel wall and has extremely high rates of mortality [1]. Early recognition of AMI can be notoriously difficult, and delayed intervention secondary to delays in diagnosis is one of the most common reasons for this extremely poor outcome [2].

Clinically, patients may present with nonspecific and vague complaints, which will lead to an extensive differential diagnosis such as abdominal pain, nausea, vomiting, anorexia, diarrhea, and obstipation. Gastrointestinal bleeding could be the primary symptom in as many as 25% of patients. When the bowel becomes necrotic, patients will present with signs of sepsis such as hypotension, fever, and tachycardia.

The severity of the pain is often disproportionate to physical examination findings, and pain could be resistant to opioids, which sometimes may be misleading and will lead the physician to suspect malingering, especially in younger patients with a history of opioid abuse. Signs of peritonitis develop late, when necrosis or perforation happens. Patients may present with severe abdominal tenderness, guarding, distension, and a tender palpable mass. Auscultation of the abdomen varies from hyperactive to absent bowel sounds.

Despite comprehensive research, no sensitive diagnostic marker for AMI has been established thus far; therefore, debates on it still continues.

This case is important because it once again emphasizes that presentation of such a detrimental, potentially fatal condition is oftentimes vague and not necessarily textbook-like and could be easily missed due to ongoing reliance upon serum lactate level, especially in patients with HIV who can have a broader differential diagnosis. Normal plasma lactate should not lower physician’s suspicion of the AMI in the right clinical setting in patients with known risk factors, such as our patient who had end-stage renal disease (ESRD) on hemodialysis (HD). Clinicians should combine their clinical impression along with appropriate imaging studies to obtain the best possible support for their suspected diagnosis and should not hesitate to administer IV contrast for patients on HD if the index of suspicion is high, as delaying the diagnosis and proper management could be life-threatening.

## Case presentation

A 35-year-old female with a medical history of HIV, ESRD on HD, hypertension (HTN), history of heparin-induced thrombocytopenia, history of multiple deep vein thromboses, and opioid dependence.

The patient presented after two weeks of abdominal pain located in her epigastrium, with an isolated episode of hematemesis. She was evaluated at a different hospital through an esophagogastroduodenoscopy (EGD), which demonstrated gastritis, and she was subsequently discharged on proton pump inhibitor. However, the day prior to her admission to our facility, she had an episode of hypotension while undergoing her scheduled dialysis. Her abdominal pain had persisted despite her recent EGD, with a new worsening of her symptoms after the episode of hypotension. Her associated symptoms included shortness of breath, dry cough, intermittent palpitations, and musculoskeletal chest wall pain. She denied any diarrhea, nauseousness, or vomiting. She was admitted to our hospital with these symptoms and quickly triaged in the emergency department. Her initial vital signs were 98° Fahrenheit on oral thermometer, heart rate of 113 beats per minute, and respiratory rate of 18 breaths per minute, and she was breathing 100% on room air. Her blood pressure ranged from 94 to 106 systolic and 40 to 49 diastolic. At the time of initial examination, her abdomen was soft, and pain was mostly reproducible on the anterior chest wall. She did not have any appreciable murmur, and her lungs were clear to auscultation.

Investigations

**Figure 1:**
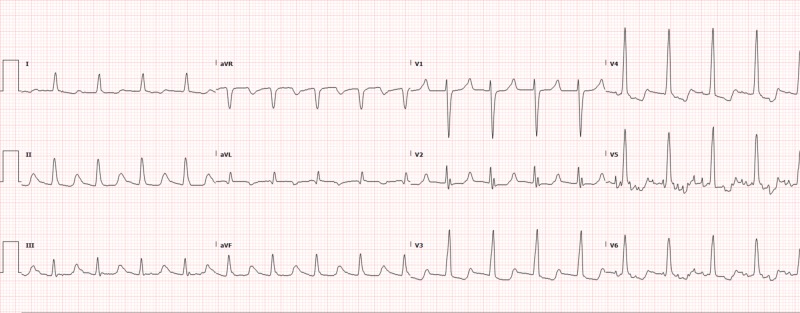
Electrocardiogram on presentation revealing sinus tachycardia and left ventricular hypertrophy with secondary re-polarization abnormalities.

Her initial CBC revealed a white blood cell (WBC) count of 15 x 10^9^/L, stable and chronic anemia with a hemoglobin of 9.4 (her baseline), and a platelet count of 237 x 10^9^/L. Her metabolic panel was unrevealing. She was also evaluated with serial troponin enzymes, which were positive at 0.17, 0.14, and later 0.12 (these were near her baseline levels in association with her chronic kidney disease). Lactic acid on admission was 1.4 mmol/L, and lipase was within normal limits. Her initial electrocardiogram (EKG) revealed sinus rhythm without any acute EKG findings (Figure 1).

Because of her immunocompromised status and duration of symptoms, she underwent imaging of the chest and abdomen with a noncontrast CT. Of note, the CT of the abdomen revealed only findings consistent with chronic constipation, and the CT of the chest revealed mild diffuse ground-glass opacities in both lungs.

Differential diagnosis

Considering her immunocompromised status and multiple comorbidities, her differential diagnosis on the first presentation was extensive. She was at risk of having an AMI (ESRD, HIV, HTN). This is what prompted evaluation through EKG and troponins. Her underlying HIV put her at risk of opportunistic infections such as pneumocystis pneumonia and hence she was evaluated with a CT of the chest. She did have evidence of ground-glass opacities, as noted on her CT, and also evidence of lymphadenopathy in her mediastinum and supraclavicular area. Hence, the consideration of underlying infection was definitely warranted. In addition, differential for her abdominal symptoms included acute pancreatitis, gastritis, intra-abdominal infections (including abscesses), and mesenteric ischemia.

Treatment

**Figure 2:**
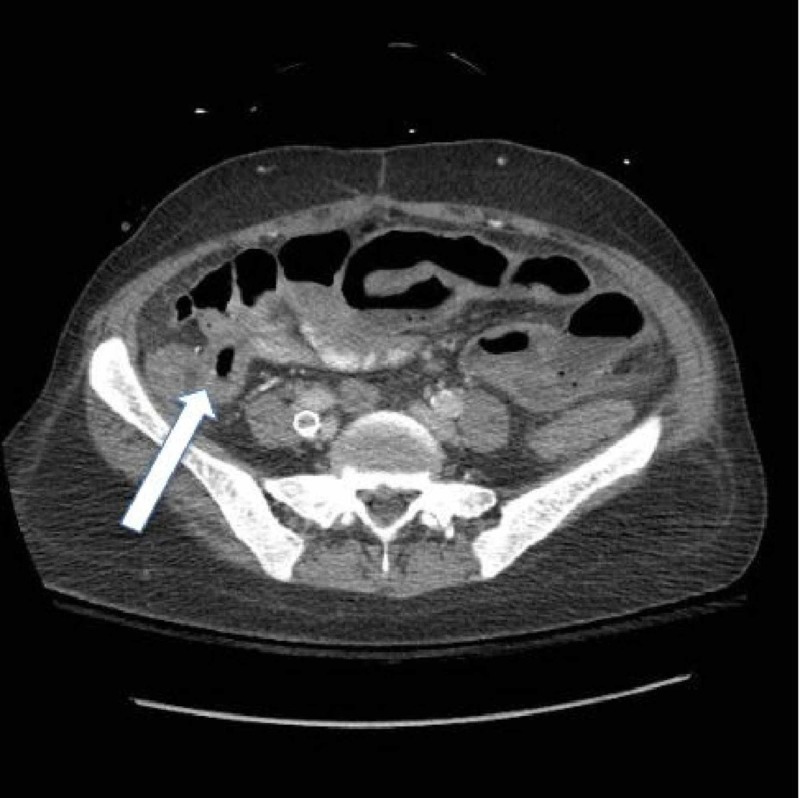
CT of the abdomen and pelvis with contrast findings showing enterocolitis involving the majority of the ileum and proximal ascending colon. These loops also demonstrate decreased enhancement worrisome for ischemia.

**Figure 3:**
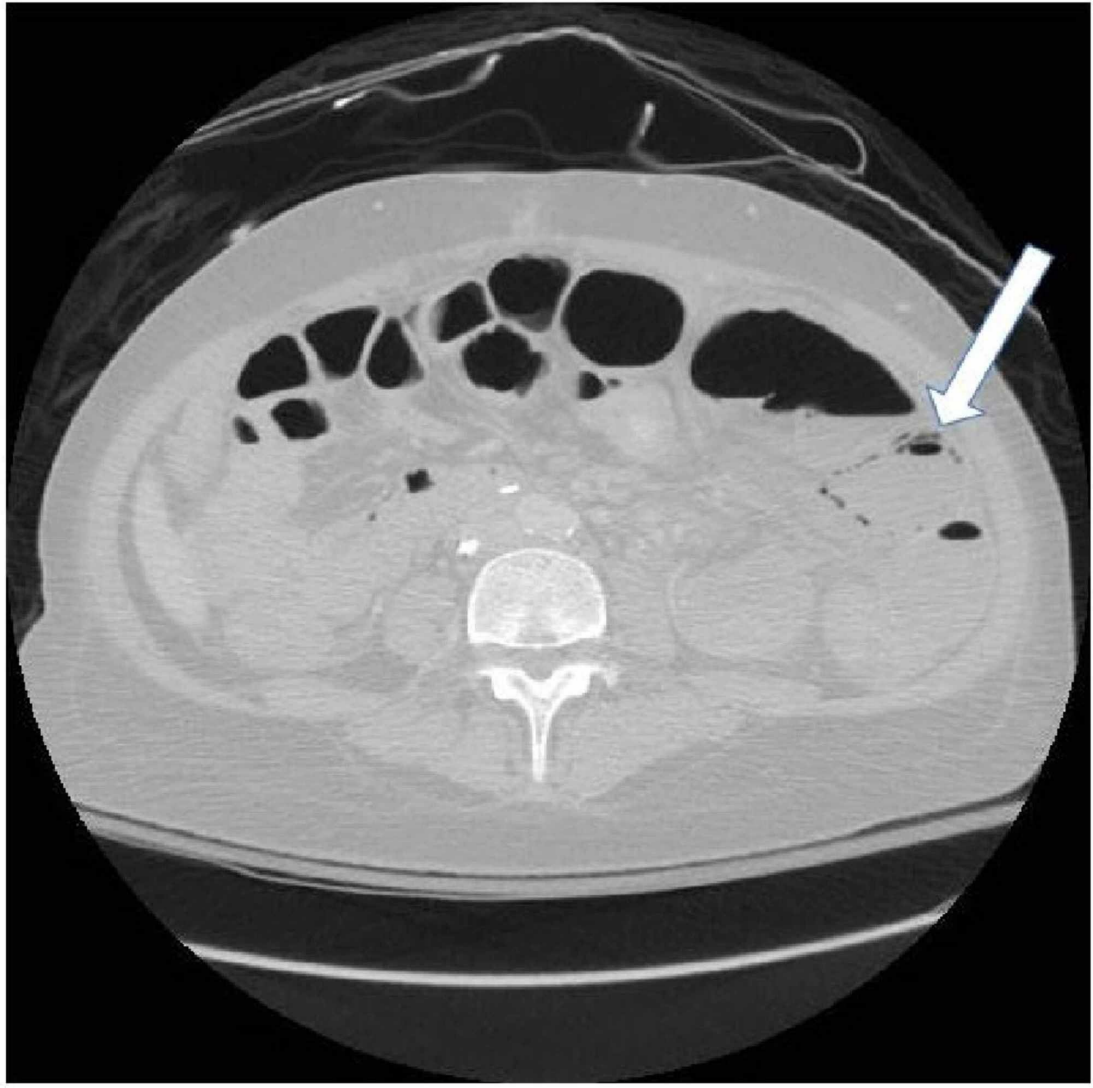
CT of the abdomen and pelvis with contrast findings showing pneumatosis involving a few borderline dilated loops of the small bowel in the lateral left mid-abdomen.

The patient was admitted to the medical unit and started on broad-spectrum antibiotics for a potential atypical infection. She, however, became hypotensive in the morning, requiring transfer to the intensive care unit and vasopressor support. She then began to spike fevers. At this point, the patient was in apparent septic shock. Her abdominal pain persisted, with the pain waxing and waning but consistently worsening. Her pain became more and more refractory to pain medication. During this time, serial lactic acid tests were performed and were all normal. Finally, due to a fear of a developing abscess or vascular compromise, a CT of the abdomen of her pelvis was repeated but now with IV contrast. This had revealed the development of enterocolitis involving the majority of the ileum and proximal ascending colon (Figure 2). There was also evidence of pneumatosis involving dilated loops of the small bowel in the lateral mid-abdomen (Figure 3). These loops demonstrated decreased enhancement concerning for ischemia.

**Figure 4:**
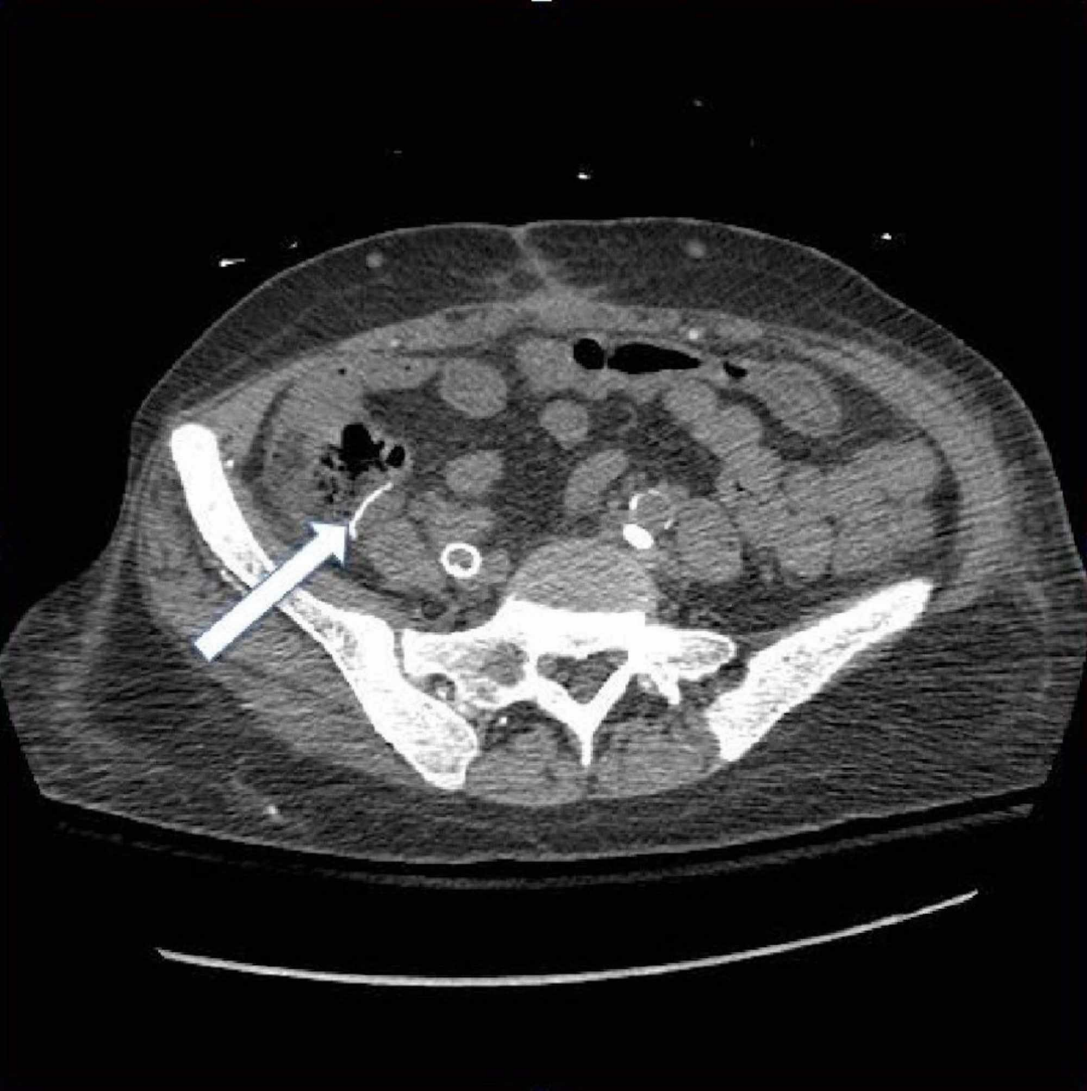
CT of the abdomen and pelvis without contrast findings showing anastomosis in the right lower quadrant, which appears to be intact with no definitive enterocolitis.

Due to these findings, general surgery was called for immediate evaluation. That same day, she underwent an exploratory laparotomy. During the operation, a large loop of the terminal ileum was necrotic and was found to immediately begin spilling succus. The bowel was necrotic, gangrene had set into the bowel wall, and there was associated perforation (Figure 4). The terminal ileum had multiple areas of patchy necrosis with signs of infection, and the terminal ileum itself (6 cm proximal to the ileocecal valve) was also necrotic and gangrenous. For these reasons, a small bowel resection was performed. Interestingly, before and after the episode of mesenteric ischemia with extensive bowel necrosis, her lactic acid had been trended and always normal.

Outcome and follow-up

After the bowel resection, the patient clinically stabilized. Her abdominal pain persisted, though more surgical in nature. Her fever trends began to decrease and she was eventually able to be transitioned off of tube feeding and began a normal diet. She was successfully weaned off vasopressors, transferred to a medical floor, and later discharged to rehabilitation.

## Discussion

Since the early 1970s, measurement of lactic acid had been considered the best serological marker for AMI. This, however, has been dispelled by several clinical and experimental studies that demonstrated that serum lactate is a nonspecific marker of tissue perfusion and fails to be an early sign during the initial phases of intestinal damage [3].

Apart from this, it is well known that the elevation of serum lactate may not be present in a significant portion of patients with AMI, as seen in our patient as well. It is also worth mentioning that existing studies could not establish a linear association between serum lactate level and the extent of bowel ischemia [2].

The study of the mechanism of lactate acid metabolism helps reveal potential causes of increased serum lactic acid besides tissue hypoperfusion. This includes impaired clearance due to hypotension, hepatic dysfunction with associated dysmetabolism, and uncoupling of oxidative phosphorylation seen with various drugs, toxins, etc. [4-5].

Jakob et al. on the dynamics of intestinal ischemia showed that intestinal hypoperfusion first results in increased lactate levels in the portal vein, which are compensated by accelerated hepatic lactate uptake and metabolization, and for the serum lactate in the general circulation to be elevated, the amount of released lactate from the ischemic gut must exceed the conversion capacity of the liver [6].

The correlation among available case studies on AMI has been variable. For instance, there are several cases in which no serum lactate acid was obtained, others in which an elevation in serum lactate was demonstrated, and a near-matching quantity of cases (like ours) in which AMI was discovered with no elevation of lactate acid being found [3].

Akutsu et al. presented a case report with type B aortic dissection with near-normal serum lactate upon admission despite intra-operatively observed overt mesenteric ischemia [7]. Another instance is seen in a retrospective study by Acosta et al. in which an elevated lactate acid level was demonstrated in less than half (12 out of the 27 patients included in the report) of the patients studied after being found to have superior mesenteric artery occlusion. [8]. Van der Voort et al. in a prospective observational cohort study of intensive care patients with clinically suspected AMI showed an increase in serum L-lactate in only 18 out of 23 patients [9]. The conclusion of the aforementioned studies is the very low specificity among patients with mesenteric ischemia, which limits the diagnostic value of serum lactate alone as a surrogate marker for bowel ischemia. Large-scale prospective studies on the alterations of lactate during AMI are very much needed.

The poor overall diagnostic value of serum lactate in the diagnosis of AMI has spurred the investigation of other potential markers of intestinal ischemia. There have been strides in the overall understanding of the pathophysiology of intestinal damage, with researchers now seeking markers specific to mucosal damage.

For example, in addition to the traditional L-lactate, the currently available serological markers of mesenteric ischemia can be classified as (1) gut lumen-derived markers such as D-lactate, (2) mucosa-derived markers such as intestinal fatty acid binding protein (i-FABP), and (3) seromuscular markers, which includes creatine kinase, lactate dehydrogenase (LDH), and glutamic oxaloacetic transaminase.

D-lactate is a lactate stereoisomer that is solely produced by bacteria in the gut lumen. AMI leads to bacterial overgrowth, which releases D-lactate into the portal and systemic circulation. Evennett et al. [10] performed a comparative analysis of all major serum markers of AMI. From all investigated markers, D-lactate demonstrated the best overall performance with the highest diagnostic accuracy index and is potentially more specific for intestinal ischemia, but elevation occurs not early enough and the medical literature still lacks comparative studies of D-lactate versus L-lactate for diagnosing AMI.

Another investigational marker of intestinal injury that has gained favor as a potential diagnostic tool for AMI is i-FABP. This protein is released from damaged enterocytes found at the tip of intestinal villi and can be detected in serum [11]. Numerous studies have detected significantly elevated i-FABP levels under AMI and pronounced promising results in terms of its specificity; however, all studies were of small size and mostly experimental in nature, and therefore clinical studies with larger cohorts and better temporal monitoring of i-FABP levels are likely to be very useful for the ultimate judgment of the role of i-FABP in AMI [12-15]. A 2016 meta-analysis (pooling from nine individual studies) demonstrated the potential diagnostic value of i-FABP, with a pooled sensitivity of 0.80 (95% CI: 0.72-0.86) and a pooled specificity of 0.85 (95% CI: 0.73-0.93) [16].

Several other markers have also been studied including creatine kinase, and elevation in WBC, C-reactive protein, LDH, amylase, alkaline phosphatase, and acid-base status at the time of diagnosis [17-18]. In general, none of these markers has proven to be superior to lactate in comparative studies [10,17-18].

It is exciting to see the development of new biochemical markers with potential promise in regard to increasing the diagnostic yield of AMI (a condition in which a delay in diagnosis is often seen with catastrophic consequences). However, there is an urgent need for validation in larger cohorts to verify diagnostic accuracy and efficiency. Until that time, CT angiography with IV contrast will still remain the gold standard and the best diagnostic tool for suspected AMI. The sensitivity (93.3 %) and specificity (95.9 %) of the CT for the diagnosis of AMI have matched the sensitivity of angiography (88 %) [19-20].

If serious clinical suspicion for AMI exists, the physician should promptly order diagnostic imaging studies without waiting for laboratory results or relying on lactate level, and an urgent abdominal CT with IV contrast is indicated to confirm the diagnosis.

## Conclusions

Here we have shown another example in which reliance on a normal lactic acid level led to a delay in the diagnosis of AMI. Serum lactic acid levels lack both sensitivity and specificity and can only aid in the detection of AMI. Normal plasma lactate does not exclude this diagnosis. In addition, though several novel biomarkers have shown potential, there is a need for further study and validation prior to widespread clinical use. Until then, AMI will remain a diagnostic challenge. This requires a physician to always have a high suspicion for this condition and use a combination of clinical impression, imaging studies, and available serological markers. Reliance on only one modality often leads to false reassurance with potentially catastrophic consequences.
